# SAMM50 Regulates Thermogenesis of Beige Adipocytes Differentiated from Human Adipose-Derived Stem Cells by Balancing Mitochondrial Dynamics

**DOI:** 10.3390/ijms23126764

**Published:** 2022-06-17

**Authors:** Se-Jun Park, Dong-Hyun Shon, Jae-Hyun Kim, Yang-Hwan Ryu, Yong Ko

**Affiliations:** 1 Division of Biotechnology, College of Life Sciences and Biotechnology, Korea University, 145, Anam-ro, Seongbuk-gu, Seoul 02841, Korea; sejun_park@korea.ac.kr (S.-J.P.); d.shon@nmslab.kr (D.-H.S.); kjah1992@korea.ac.kr (J.-H.K.); brain1905@biosolutions.co.kr (Y.-H.R.); 2 NMS LAB, 311, Anyang-ro, Manan-gu, Anyang-si 14001, Korea; 3 R&D Institute, Biosolution Co., Ltd., 232, Gongneung-ro, Nowon-gu, Seoul 01811, Korea

**Keywords:** adipose-derived stem cells, thermogenic adipocytes, mitochondrial dynamics, obesity, SAMM50, UCP1

## Abstract

Brown/beige adipocyte thermogenesis is a process that is important for energy balance. The thermogenesis of brown/beige adipocytes occurs in the mitochondria, which is modulated by the dynamic balance between mitochondrial fusion and fission. Mitophagy is also involved in mitochondrial dynamics. The sorting and assembly machinery (SAM) complex protein, SAMM50, plays a key role in mitochondrial dynamics and quality control through regulating mitophagy. However, the roles of SAMM50 in the thermogenesis of beige adipocytes remain unknown. Thus, the objective of this study was to conduct functional analyses of SAMM50. The expression of mitochondrial fusion genes was repressed by SAMM50 knockdown but was not altered by SAMM50 overexpression. These results agreed with the distribution of the fluorescence-stained mitochondria and an mtDNA copy number. In contrast, the expression of mitochondrial fission genes showed an opposite outcome. As a result, suppression by the SAMM50 shRNA inhibited the expression of thermogenic genes (*UCP1*, *PPARGC1A*, *DIO2*, *ELOVL3*, *CIDEA*, and *CIDEC*) and mitochondrial-related genes (*CYCS*, *COX7A1*, *TFAM*, *CPT1B*, and *CPT2*). Conversely, SAMM50 overexpression promoted the expression of the thermogenic genes and mitochondrial genes. Thus, SAMM50 links the balance between the mitochondrial dynamics and thermogenesis of beige adipocytes.

## 1. Introduction

In mammals, adipose tissues are classified into three types: white adipose tissues (WAT), brown adipose tissues (BAT), and brown-like (beige or brite) adipose tissues. Each is composed of diverse morphological and physiological adipocyte communities [1]. WAT generally stores energy as triglycerides (TG), whereas the BAT and beige adipose tissues burn energy by uncoupling protein 1 (UCP1)-mediated thermogenesis. Morphologically, white adipocytes have single large lipid droplets and a small number of mitochondria, while the brown and beige adipocytes have multilocular lipid droplets and large numbers of mitochondria. Both the brown and beige adipocytes express UCP1, a key thermogenic marker. They are physiologically and morphologically similar [1,2]. However, they can be distinguished into different cell types based on their developmental properties. The brown adipocytes develop from Myf5-positive progenitors, whereas the beige adipocytes form in WAT depots and derive from a variety of origins depending on the depot [3,4]. In particular, beige adipocytes can be activated within many WAT depots by cold exposure or β-adrenergic stimuli, thus exhibiting a higher degree of thermogenic plasticity. This process is known as browning (or beiging) [5,6,7]. Because BAT is rarely present in adult humans, the browning of the white adipocytes could be an attractive strategy to combat obesity and metabolic disorders [8].

UCP1-mediated thermogenesis can uncouple the ATP synthesis and control the proton concentration, producing heat in mitochondrial respiration [2]. The presence of UCP1 in the inner mitochondrial membrane reveals that mitochondria are essential organelles in beige adipocytes [6]. These organelles go through a sequence of dynamic processes, including degradation, biogenesis, fusion, and fission. Mitochondrial biogenesis increases mitochondrial volume density, as observed in BAT and the browning of WAT [9]. Mitochondrial fusion and fission can regulate mitochondrial morphology and quality [10]. Fusion is associated with mitofusins (MFN1 and MFN2) and optic atrophy 1 (OPA1), whereas fission is regulated by dynamin-related protein 1 (DRP1). MFN2 deletion in adipose tissues can reduce thermogenesis, however, it can protect against a high-fat diet [11,12]. In addition, it has been reported that OPA1 can promote the browning of WAT [13]. The dynamic modification of mitochondrial shape and morphology can affect mitochondrial function. In patients with obesity and type 2 diabetes (T2D), it has been observed that mitochondrial fusion is decreased, but that fission is increased, indicating that an abnormal mitochondrial morphology is linked to the development of obesity-related metabolic disorders [14,15]. As such, it is very important to balance the mitochondrial biogenesis and dynamics in the thermogenesis of beige adipocytes.

Mitochondrial degradation is promoted by mitophagy, a type of autophagy. Mitophagy plays a key role in mitochondrial quality control, which involves the degradation of defected or redundant mitochondria through a selective pathway [16,17]. When thermogenic stimuli are removed, beige adipocytes activate mitophagy and return to a white adipocyte phenotype [18,19]. Mitophagy is initiated and regulated by PTEN-induced kinase 1 (PINK1) and Parkin in defected or redundant mitochondria [20]. The accumulation of PINK1 in the outer membrane of mitochondria (OMM) induces the phosphorylation of Parkin and results in the ubiquitination of OMM proteins, leading to mitophagy [21,22]. There are several translocases in OMM, including the channel-forming protein, TOM40, for protein transport into mitochondria, and the sorting and assembly machinery (SAM) for membrane integration and assembly [23]. A member of the SAM complex, SAMM50, is essential for mitochondrial membrane organization and respiratory complex assembly, regulating cristae stability by interacting with the mitochondrial contact site and cristae organizing system complex [24,25,26]. Recent studies have reported that SAMM50 is a key regulator of mitochondrial dynamics and mitophagy, which is mediated by PINK1-Parkin through interactions with PINK1 [27,28].

However, its role in the thermogenesis of human-beige adipocytes remains unknown. Based on the beige adipocyte physiology, this study hypothesizes that SAMM50 has a positive role in the thermogenesis of beige adipocytes by regulating mitochondrial dynamics. Therefore, the purpose of this study was to investigate the physiological role of SAMM50 in thermogenesis and mitochondria in the established human-beige adipocytes.

## 2. Results

### 2.1. Characterization of Adipogenic Differentiation and SAMM50 Expression of Beige Adipocytes

**Figure 1:**
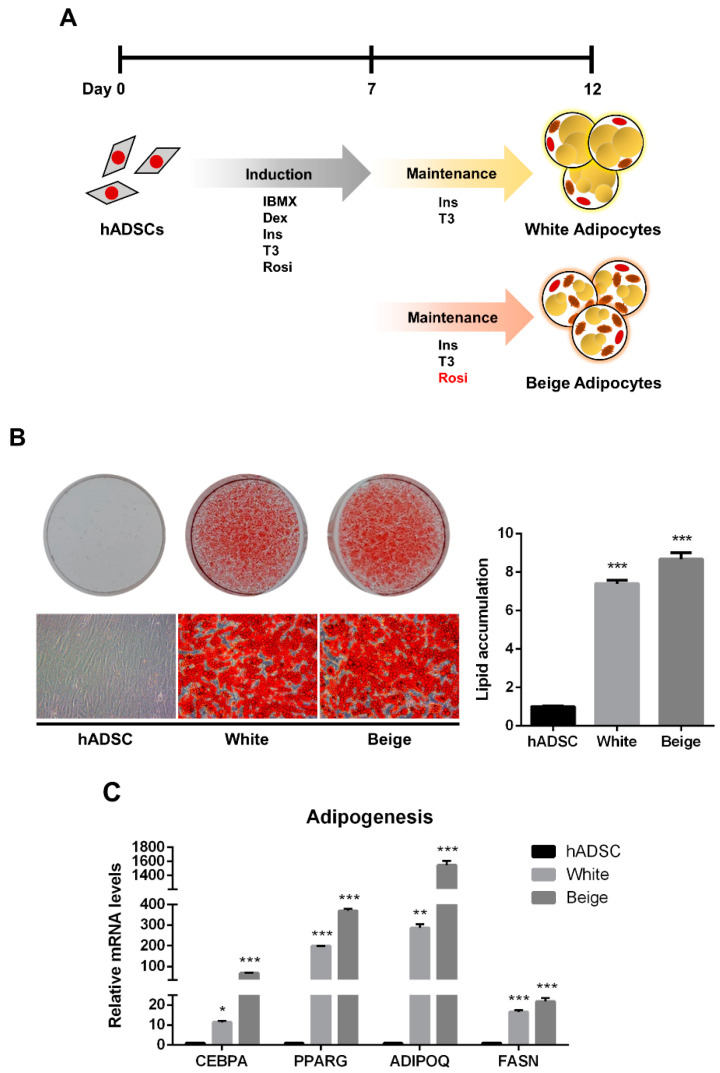
The adipogenic differentiation of human adipose-derived stem cells. (**A**) Schematic images of human adipose-derived stem cell differentiation into white and beige adipocytes. (**B**) The cells were stained with Oil Red O. The lipid accumulation levels were measured by absorbance at 490 nm by extracting Oil Red O dye with isopropanol. (**C**) The expression levels of adipogenic genes were assayed by qPCR. Results are shown as mean ± SEM. * *p* < 0.05; ** *p* < 0.01; *** *p* < 0.005 compared to the hADSC group.

**Figure 2:**
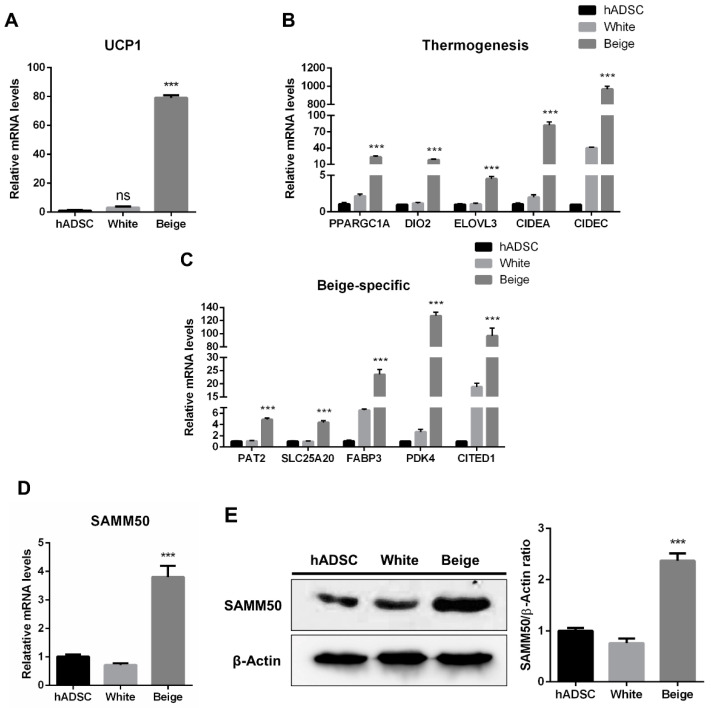
The establishment of beige adipocytes differentiated from human adipose-derived stem cells and SAMM50 expression in beige adipocytes. (**A**) qPCR analysis assessed *UCP1* mRNA levels in the human adipose-derived stem cells and differentiated cells. (**B**,**C**) The expression levels of thermogenic (**B**) and beige-specific (**C**) genes were investigated by qPCR. (**D**,**E**) qPCR and Western blot analyses were performed to examine mRNA and protein levels of SAMM50 in the human adipose-derived stem cells and the differentiated cells. The protein levels of SAMM50 were quantified using ImageJ software. Results are shown as mean ± SEM. *** *p* < 0.005 compared to the hADSC and white group.

The human adipose-derived stem cells (hADSCs) were differentiated into white and beige adipocytes by treating them with an adipogenic cocktail for 12 days (Figure 1A). Oil Red O (ORO) staining showed significantly increased lipid accumulation in the differentiated groups than in the hADSC group (Figure 1B, *p* < 0.005). Additionally, the expression levels of adipogenic markers were significantly elevated in the beige and white groups compared with the hADSC group (Figure 1C, *p* < 0.05). To determine whether beige adipocytes were properly differentiated, a beige adipogenic marker, *UCP1*, was investigated, and it was highly expressed in the beige group compared to the hADSC group and the white group (Figure 2A and Figure S1, *p* < 0.005). Thermogenic markers, including *PPARGC1A*, *DIO2*, *ELOVL3*, *CIDEA*, and *CIDEC* were expressed significantly higher in the beige group compared with the hADSC group and the white group (Figure 2B, *p* < 0.005). Moreover, the expression levels of beige-specific markers, including *PAT2*, *SLC25A20*, *FABP3*, *PDK4*, and *CITED1*, were also higher in the beige group compared to the hADSC and white groups (Figure 2C, *p* < 0.005). In particular, the SAMM50 mRNA and protein levels were significantly higher in the beige group compared with both the hADSC and white groups (Figure 2D,E, *p* < 0.005).

### 2.2. The Expression of Mitochondrial Dynamics Genes Is Regulated by Modulating the Expression of SAMM50 in Beige Adipocytes

**Figure 3:**
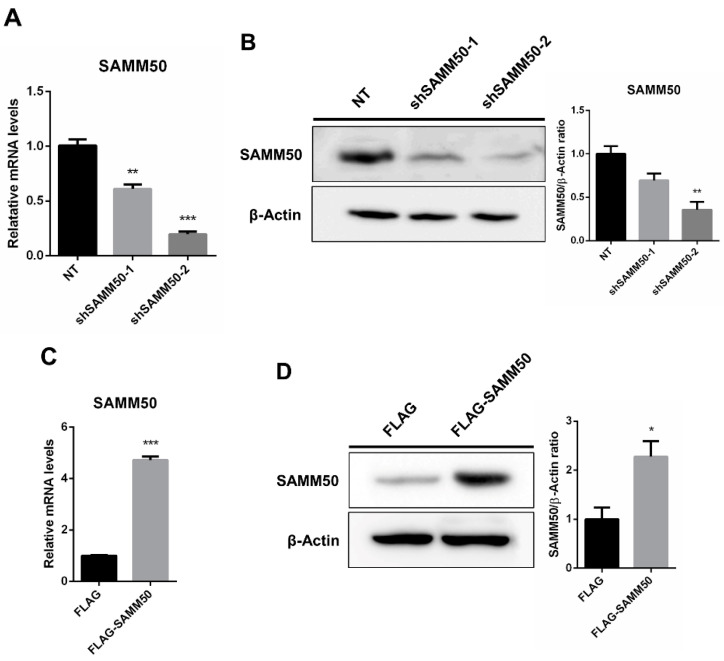
Knockdown and overexpression of SAMM50 in human adipose-derived stem cells. (**A**,**B**) qPCR and Western blot analyses were performed to determine mRNA and protein levels of SAMM50 in the shSAMM50-infected human adipose-derived stem cells using lentivirus. (**C**,**D**) qPCR and Western blot analyses were performed to assess mRNA and protein levels of SAMM50 in the human adipose-derived stem cells infected with FLAG-tagged SAMM50 using lentivirus. The protein levels of SAMM50 were quantified using ImageJ software. Results are shown as mean ± SEM. * *p* < 0.05; ** *p* < 0.01; *** *p* < 0.005 compared to the controls (NT and FLAG group, respectively).

**Figure 4:**
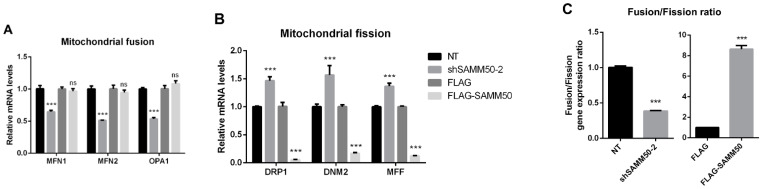

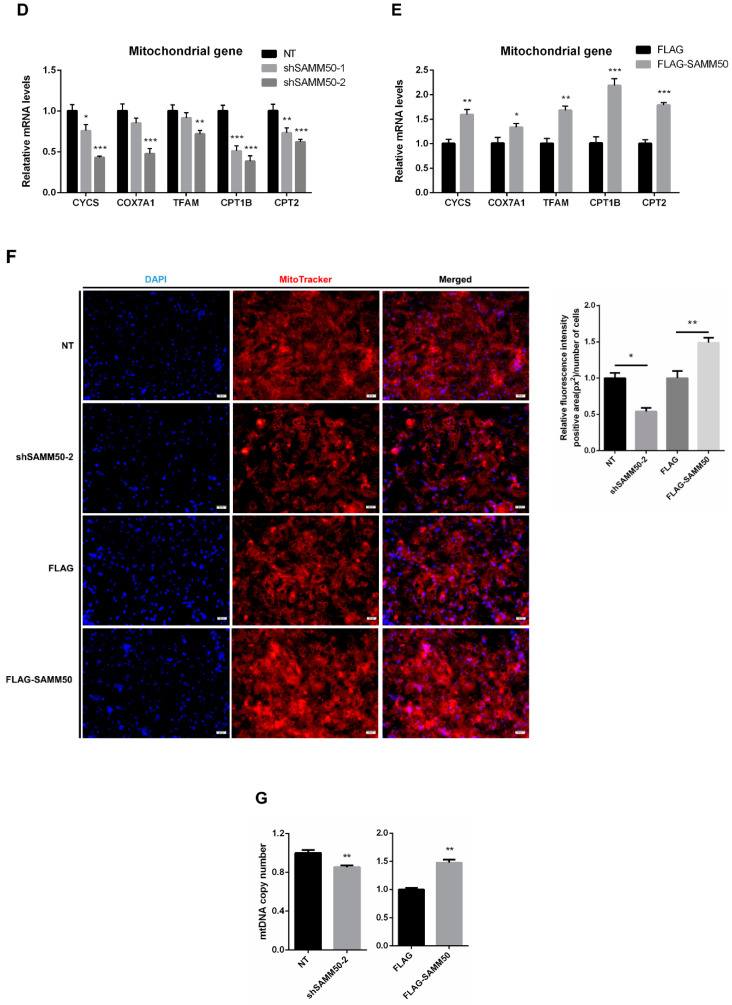
Effects of SAMM50 knockdown and overexpression on the mitochondrial dynamics and biogenesis of beige adipocytes. (**A**,**B**) Relative mRNA levels of mitochondrial fusion (**A**) and fission (**B**) genes in beige adipocytes infected with shSAMM50 and FLAG-tagged SAMM50 were analyzed by qPCR. (**C**) The expression ratio of fusion/fission was calculated as the ratio of fusion genes to fission genes. (**D**,**E**) The expression levels of mitochondrial genes in beige adipocytes infected with shSAMM50 (**D**) and FLAG-tagged SAMM50 (**E**) were assayed by qPCR. (**F**) Representative fluorescence images of beige adipocytes infected with shSAMM50 and FLAG-tagged SAMM50 were stained with MitoTracker (red). The fluorescence levels of MitoTracker were quantified using ImageJ software. (**G**) Relative mtDNA copy numbers in beige adipocytes infected with shSAMM50 and FLAG-tagged SAMM50 were investigated by qPCR. Results are shown as mean ± SEM. * *p* < 0.05; ** *p* < 0.01; *** *p* < 0.005 compared to the controls.

The regulatory action of SAMM50 in the beige adipocyte’s physiology was analyzed using the short hairpin RNA (shRNA) and expression vectors. Firstly, the knockdown of SAMM50 inhibited both the mRNA and protein levels of SAMM50 in the infected hADSCs. Especially, the expression of SAMM50 was significantly suppressed in the shSAMM50-2 group compared to the NT group (Figure 3A,B, *p* < 0.01). Meanwhile, the FLAG-tagged SAMM50, which was infected in hADSCs with the use of a lentivirus, significantly increased the SAMM50 mRNA and protein levels in the FLAG group (Figure 3C,D, *p* < 0.05). Additionally, the protein level of FLAG was evaluated, confirming the overexpression of FLAG protein in the FLAG-SAMM50 group (Figure S2, *p* < 0.01). In order to identify the role of SAMM50 in the mitochondria of beige adipocytes, the expression levels of the mitochondrial dynamics-related genes were analyzed. These results showed that the expression levels of the mitochondrial fusion markers, including *MFN1*, *MFN2*, and *OPA1* were significantly down-regulated in the shSAMM50-2 group compared with the NT group (*p* < 0.005), however, there was no significant difference shown in the FLAG-SAMM50 group compared with the FLAG group (Figure 4A). On the contrary, the expression levels of the mitochondrial fission regulatory factors *DNM1L*, *DNM2*, and *MFF* were significantly promoted in the shSAMM50-2 group, however, they were inhibited in the FLAG-SAMM50 group when compared to the NT group and the FLAG group, respectively (Figure 4B, *p* < 0.005). As a result, it was shown that the expression ratio of the fusion/fission genes activated fission in knockdown and fusion in overexpression, according to the expression change in SAMM50 (Figure 4C, *p* < 0.005).

### 2.3. SAMM50 Regulates Mitochondrial Biogenesis in Beige Adipocytes

To demonstrate whether regulated thermogenesis in beige adipocytes was due to SAMM50 expression, mitochondrial biogenesis was investigated. The mitochondrial markers, *CYCS*, *COX7A1*, *TFAM*, *CPT1B*, and *CPT2* were inhibited in the shSAMM50-2 group, whereas up-regulated in the FLAG-SAMM50 group (Figure 4D,E, *p* < 0.01). As SAMM50 regulated the expression of the mitochondrial genes in beige adipocytes, MitoTracker and mtDNA copy number analyses were performed to determine whether SAMM50 controlled the mitochondrial content in beige adipocytes. Fluorescence-stained mitochondria with MitoTracker were decreased in the shSAMM50-2 group, however, they increased in the FLAG-SAMM50 group when compared to that in the NT group and the FLAG group, respectively (Figure 4F). The same tendency, in regards to the mtDNA copy number, was also observed (Figure 4G, *p* < 0.05).

### 2.4. SAMM50 Regulates Thermogenic Factors in Beige Adipocytes

**Figure 5:**
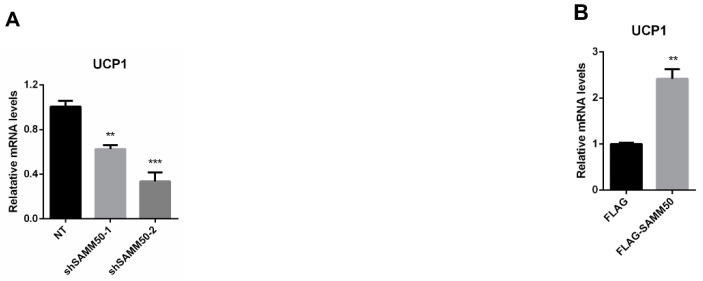

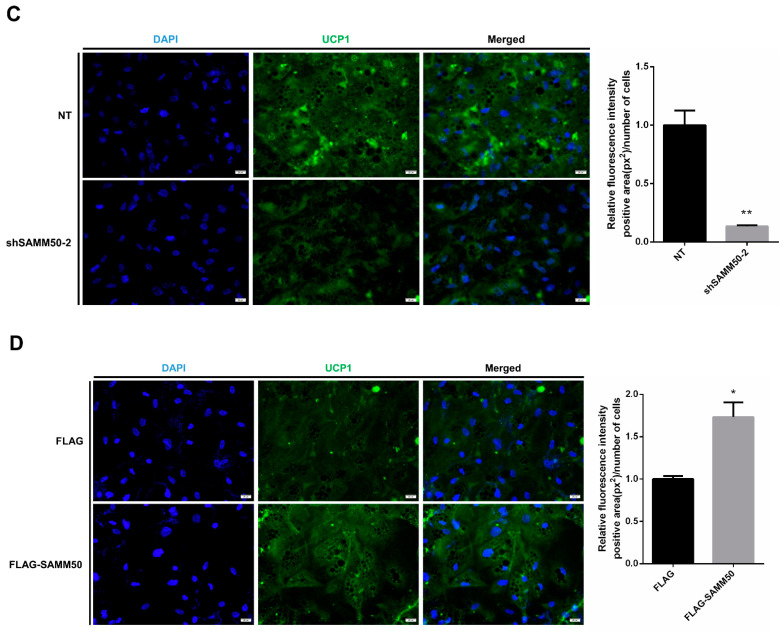
Effects of SAMM50 knockdown or overexpression on the expression of UCP1 in beige adipocytes. (**A**,**B**) The expression levels of *UCP1* in beige adipocytes infected with shSAMM50 (**A**) and FLAG-tagged SAMM50 (**B**) were analyzed using qPCR. (**C**,**D**) Representative fluorescence images of beige adipocytes infected with shSAMM50 (**C**) and FLAG-tagged SAMM50 (**D**) were stained with DAPI (blue) and anti-UCP1 (green), respectively. The fluorescence levels of UCP1 were quantified using ImageJ software. Results are shown as mean ± SEM. * *p* < 0.05; ** *p* < 0.01; *** *p* < 0.005 compared to the controls.

**Figure 6:**
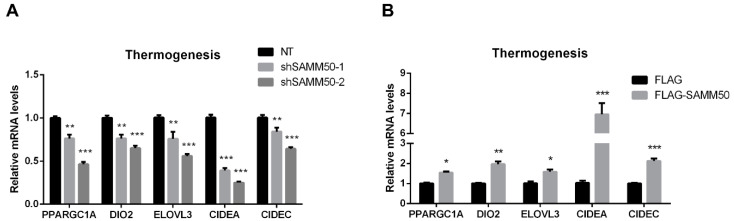

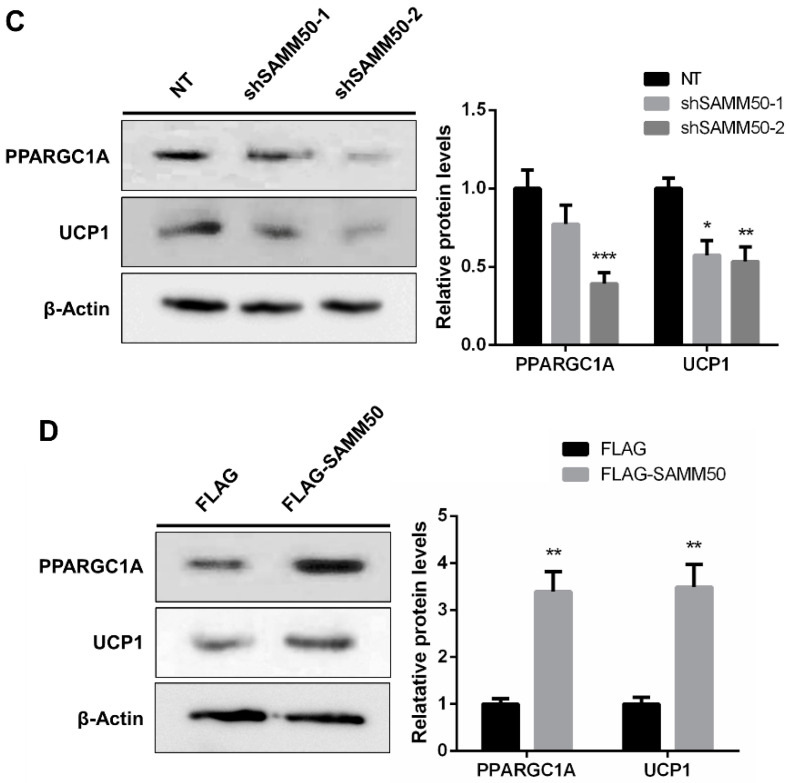
The effects of SAMM50 knockdown and overexpression on the thermogenesis of beige adipocytes. (**A**,**B**) The expression levels of thermogenic genes in beige adipocytes infected with shSAMM50 (**A**) and FLAG-tagged SAMM50 (**B**) were analyzed by qPCR analysis. (**C**,**D**) Western blot analysis was performed to determine the protein levels of PPARGC1A and UCP1 in the shSAMM50 (**C**) and FLAG-tagged SAMM50 (**D**) -infected beige adipocytes. The protein levels of PPARGC1A and UCP1 were quantified using ImageJ software. Results are shown as mean ± SEM. * *p* < 0.05; ** *p* < 0.01; *** *p* < 0.005 compared to the controls.

The expression of *UCP1* was significantly reduced in the shSAMM50 groups compared with the NT group (Figure 5A, *p* < 0.01). In contrast, the mRNA level of *UCP1* was significantly up-regulated in the FLAG-SAMM50 group compared with the FLAG group (Figure 5B, *p* < 0.01), along with a decreased intracellular UCP1 in the shSAMM50-2 group but an increase in the FLAG-SAMM50 group (Figure 5C,D). The expression levels of thermogenic genes, including *PPARGC1A*, *DIO2*, *ELOVL3*, *CIDEA*, and *CIDEC* were down-regulated by SAMM50 knockdown (Figure 6A, *p* < 0.005). On the other hand, the overexpression of SAMM50 up-regulated the expression of these thermogenic genes in beige adipocytes (Figure 6B, *p* < 0.05). Furthermore, the protein levels of PPARGC1A and UCP1 were significantly suppressed in the shSAMM50-2 group when compared to the NT group (Figure 6C, *p* < 0.01), while the overexpression of SAMM50 elevated the protein levels of PPARGC1A and UCP1 in the FLAG-SAMM50 group when compared to the FLAG group (Figure 6D, *p* < 0.01).

## 3. Discussion

The thermogenesis of brown/beige adipocytes plays an important role in the controlling energy balance in the treatment of obesity and metabolic disorders [8,29]. The location of UCP1 in the inner mitochondrial membrane suggests that the mitochondrion is an important cytoplasmic organelle for the thermogenesis of brown/beige adipocytes [6]. Mitochondrial properties exert a reversible effect on the metabolism and energy balance of cells overall. Therefore, quality control, regulated through the mitochondrial dynamics of movement, fusion, and fission, is essential [30]. Moreover, mitochondrial dynamics are reported to play a role in maintaining electrical and biochemical connectivity, modulating metabolic efficiency, protecting mitochondrial DNA, regulating apoptosis, and interacting with other cellular organelles [30]. Additionally, mitochondrial morphology acts as the consequence of a balance between fission and fusion activities [30], and it has been reported that the regulation of morphology and energy metabolism through mitochondrial dynamics is related to the thermogenesis of brown/beige adipocytes [31]. The mitochondrial dynamics that regulate thermogenesis involve not only the respiratory complex assembly and production of ROS, but additionally, the modulation of mitochondria interaction with intracellular organelles (endoplasmic reticulum and lipid droplets) [31,32]. Therefore, several studies have shown that the balancing of the mitochondrial dynamics has the potential to regulate the thermogenesis of beige adipocytes [31].

Generally, beige adipocytes are characterized by the high expression of UCP1 [2], and additionally, the gene expression of *PPARGC1A*, *DIO2*, *ELOVL3*, *CIDEA*, and *CIDEC* has been reported as a biomarker for thermogenic activity [2,6]. Particularly, PPARGC1A acts as a transcriptional key regulator of thermogenesis through the regulation of mitochondrial biogenesis, which is involved in the initialization of adipocyte browning [3]. As biomarkers determine beige adipocytes, the expression of genes, such as *PAT2*, *SLC25A20*, *FABP3*, *PDK4*, and *CITED1* has been observed [2,3,33,34]. In this study, the differentiation of hADSCs into beige adipocytes was established. Additionally, lipid accumulation and adipogenic markers, *CEBPA*, *PPARG*, *ADIPOQ*, and *FASN* were significantly increased in both the white and beige adipogenic differentiation of hADSCs when compared with the undifferentiated hADSCs (Figure 1B,C). Similarly, the expression of thermogenic and beige-specific genes, including UCP1, significantly increased in the beige group when compared with the white and hADSC group, confirming the differentiation of hADSCs into beige adipocytes (Figure 2A,C).

Another characteristic feature of beige adipocytes is their active metabolic function through a high mitochondrial concentration [6]. Specifically, mitophagy can induce mitochondrion degradation through PINK1-Parkin-mediated autophagy [20]. Moreover, recent studies have shown that mitophagy is involved in the conversion of beige adipocytes to white adipocytes in humans [19]. Recently, SAMM50 has been reported as a key regulator of PINK1-Parkin-mediated mitophagy and mitochondrial dynamics [28]. Based on all of these findings, it was hypothesized that SAMM50 could regulate the mitochondrial dynamics in the thermogenesis of beige adipocytes. The significantly elevated levels of SAMM50 mRNA and protein in the established beige adipocytes (Figure 2D,E) were corresponding to its function analyzed by the lentiviral system (Figure 3). Either the suppression or overexpression of SAMM50 was correlated with the expression profile of thermogenic factors, including UCP1 and PPARGC1A (Figure 5 and Figure 6), indicating that SAMM50 regulates the thermogenesis of differentiated beige adipocytes from hADSCs.

SAMM50 is essential for mitochondrial membrane organization and respiratory complex assembly and regulates the cristae stability through an interaction with the mitochondrial contact site and the cristae organizing system complex [25,26]. According to a recent study, SAMM50 can bind to the PINK1 and mitochondrial dynamic factors, including MFN1, MFN2, and MFF and, SAMM50 deficiency causes PINK1-Parkin-mediated activity [28]. This report indicates that SAMM50 plays an important role in mitochondrial dynamics and quality control and that SAMM50 inhibits PINK1-Parkin-mediated mitophagy. Additionally, it has been reported that PINK1-Parkin-mediated mitophagy is highly associated with mitochondrial dynamics [35]. Mitophagy is facilitated through either the increased fission or decreased fusion of mitochondria, whereas it is inhibited by an increased fusion or decreased fission [36]. In this study, the expression levels of the mitochondrial fusion genes, including *MFN1*, *MFN2*, and *OPA1*, were decreased in SAMM50 knockdown, however, no change was observed in SAMM50 overexpression (Figure 4A). Furthermore, the expression levels of the mitochondrial fission genes, including *DRP1*, *DNM2*, and *MFF*, were promoted in SAMM50 knockdown, while they were suppressed in SAMM50 overexpression (Figure 4B). In the knockdown of SAMM50, the mitophagy was promoted by a decreased fusion and increased fission, which is consistent with previous studies. However, in the overexpression of SAMM50, the fusion remained unchanged, but the fission was strongly suppressed, exhibiting results opposite to the previous study with HeLa cells [36]. These results are assumed to be characteristic of beige adipocytes having unique functions and morphology with different mitochondrial populations [37]. The results obtained in this study indicate that the expression of SAMM50 regulates the balance of mitochondrial fusion/fission. In addition, the expression levels of mitochondrial-related genes, including *CYCS*, *COX7A1*, *CPT1B*, and *CPT2*, were all inhibited by the inhibition of SAMM50, whereas it was all elevated in the overexpression of SAMM50 (Figure 4D,E). To confirm this role, the MitoTracker staining revealed that the mitochondrial content fluctuated when the expression of the SAMM50 changed, with the mtDNA copy number showing a consistent trend (Figure 4F,G). It was postulated that the number of mitochondria decreased as mitochondrial degradation was promoted through the inhibition of the fusion gene and the promotion of the fission gene in SAMM50 knockdown. These results are thought to increase mitochondrial biogenesis by inhibiting mitophagy as SAMM50 regulates fusion/fission. As a result, in the thermogenesis of beige adipocytes, the expression of SAMM50 was positively correlated with the expression profile of thermogenic factors, including UCP1 and PPARGC1A (Figure 5 and Figure 6), indicating that SAMM50 could regulate the thermogenesis of differentiated beige adipocytes from hADSCs. Thus, these findings demonstrate that, along with the mitochondrial-related genes, thermogenic genes are also regulated in accordance with the mitochondrial content that is regulated through the expression of SAMM50. Especially, SAMM50, in these roles, may be involved in improving obesity. The thermogenesis of beige adipocytes induces energy consumption with a higher degree of thermogenic plasticity, thus contributing to the treatment of energy-accumulation-induced obesity [7]. In addition, it has been reported that the mitochondrial quality control mechanisms, including the mitochondrial dynamics of beige adipocytes, effectively regulate thermogenesis as well as ameliorate several metabolic disorders [14]. In this study, the SAMM50-induced mitochondrial quality control of beige adipocytes could serve as a counter to obesity by promoting thermogenesis.

In conclusion, an altered expression of SAMM50 in human-beige adipocytes affects the mtDNA and mitochondrial biogenesis through the regulation of mitochondrial fusion/fission gene expression, which leads to the regulation of mitochondria-related thermogenesis of beige adipocytes. Therefore, these results demonstrate that SAMM50 regulates mitochondrial biogenesis and controls the thermogenesis of differentiated beige adipocytes from hADSCs via controlling the balance of mitochondrial dynamics. This study suggests that SAMM50 has a potential role in obesity and related metabolic disorders through the thermogenesis of beige adipocytes by targeting mitochondrial dynamics.

## 4. Materials and Methods

### 4.1. Cell Culture and Differentiation

The hADSCs were obtained from Biosolution Co., Ltd. (Korea) and grown in a 1:1 mixture of Dulbecco’s Modified Eagle’s Medium and Ham’s F-12 (DMEM/F12) containing 1% penicillin/streptomycin and 10% fetal bovine serum (FBS) (all from Thermo Fisher Scientific, Waltham, MA, USA) at 37 °C in a humidified atmosphere with 5% CO_2_. For the white and beige adipogenic differentiation, the cells were cultured for 48 h after becoming fully confluent and were then induced to differentiate in an induction medium supplemented with 0.5 mM isobutylmethylxanthine (IBMX), 100 nM dexamethasone, 1 µM of rosiglitazone, 2 nM 3 3′5-triiodo-L-thyronine (T3), and 100 nM insulin (Sigma-Aldrich, St. Louis, MI, USA) for 7 days. They were then further cultured for 5 days until adipogenic differentiation was completed in the maintenance medium supplemented with 2 nM T3 and 100 nM insulin. For the beige adipocyte differentiation, it was induced by adding 1 µM of rosiglitazone to the maintenance medium.

### 4.2. Quantitative Reverse Transcription PCR

The total RNA was prepared from cultured cells (hADSCs at day 0, white and beige adipocytes at day 12) using TRIzol (Thermo Fisher Scientific, Waltham, MA, USA), and was quantified using Nanodrop, ND-100 (Thermo Fisher Scientific, Waltham, MA, USA), whereby 1 µg of total RNA was then synthesized to cDNA using a cDNA synthesis kit (CellSafe, Yongin, Gyeonggi, Korea), according to the manufacturer’s instructions. Quantitative PCR (qPCR) was performed with a Step One Plus Real-Time PCR system (Thermo Fisher Scientific, Waltham, MA, USA) using a Premier qPCR kit (NanoHelix, Daejeon, Korea), and the specific sequences of primers used in this study are described in Table S1.

### 4.3. Western Blot Analysis

The differentiated white and beige adipocytes, for 12 days, were lysed in a radio-immunoprecipitation assay (RIPA) lysis buffer supplemented with a protease inhibitor cocktail (Bio Basic Inc., Markham, ON, Canada). Cell lysates (20 µg) were then separated by 12% sodium dodecyl sulfate–polyacrylamide gel electrophoresis (SDS-PAGE) and then transferred to polyvinylidene fluoride (PVDF) membranes (GE Healthcare, Chicago, IL, USA). The membranes were blocked with 3% skim milk in tris-buffered saline with 0.1% tween 20 (TBS-T) buffer and probed with primary antibodies against β-Actin (SC47778) (Santa Cruz Biotechnology, Dallas, TX, USA), PPARGC1A (A12348), SAMM50 (A3401), and UCP1 (A5857) (ABclonal Inc., Woburn, MA, USA). The membranes were then washed with TBS-T buffer and probed with horseradish peroxidase (HRP)-conjugated secondary antibodies (SC2004) (Santa Cruz Biotechnology, Dallas, TX, USA). The proteins were detected through an enhanced chemiluminescence system (ATTO, Amherst, NY, USA) using an ImangeQuant LAS 4000 mini (GE Healthcare, Chicago, IL, USA), and the relative protein levels were quantified with the ImageJ program (NIH, Bethesda, MD, USA).

### 4.4. Plasmid Construction and Lentivirus Transduction

Two individual *SAMM50* shRNA target sequences were selected from the Genetic Perturbation Platform (GPP) (Table S2) and then cloned into a commercial pLKO.1-puro lentiviral vector (AgeI/EcoRI) (gifted from David Root, Addgene plasmid #10879; http://n2t.net/addgene:10879; RRID:Addgene_10879) [38], and the non-targeting shRNA (NT) pLKO.1 vector (gifted by David Sabatini, Addgene plasmid #1864; http://n2t.net/addgene:1864; RRID:Addgene_1864) was used as a control [39]. The HEK293FT cells (Thermo Fisher Scientific, Waltham, MA, USA) in 100 mm plates were transfected with 1 µg of pLKO.1-NT-puro, 1 µg of pLKO.1-shSAMM50-puro, and the lentiviral packaging vectors (3 µg of CMV delta 8.9 and 4 µg of VSV-G), respectively, using Lipofectamine 2000 (Thermo Fisher Scientific, Waltham, MA, USA) according to the manufacturer’s protocol, and the lentiviral packaging vectors were kindly provided by Dr. Hyunggee Kim (Korea University, Seoul, Korea). The lentiviral medium for shSAMM50 was harvested at 48 h after transfection. The hADSCs were infected with lentiviral supernatants in the presence of 6 µg/mL of polybrene (Sigma-Aldrich, St. Louis, MI, USA). The infected hADSCs were selected with 2 µg/mL of puromycin (Sigma-Aldrich, St. Louis, MI, USA) over 7 days.

For the overexpression, human *SAMM50* cDNA was obtained through PCR amplification and cloned into a pLL-EF1α-FLAG-PGK-BSD, as described previously [40], with the plasmid kindly provided by Dr. Hyunggee Kim (Korea University, Seoul, Korea). The HEK293FT cells were transfected with 1 µg of pLL-EF1α-FLAG-PGK-BSD, 1 µg of pLL-EF1α-FLAG-SAMM50-PGK-BSD, and the lentiviral packaging vectors (3 µg of CMV delta 8.9 and 4 µg of VSV-G) using Lipofectamine 2000 (Thermo Fisher Scientific, Waltham, MA, USA) according to the manufacturer’s protocol. The lentiviral medium for FLAG-SAMM50 was harvested at 48 h after transfection, and the infected hADSCs were selected with 4 µg/mL of blasticidin S (Sigma-Aldrich, St. Louis, MI, USA) over 14 days.

### 4.5. Oil Red O Staining

The differentiated white and beige adipocytes, for 12 days, were washed with PBS and fixed with 4% paraformaldehyde at room temperature for 1 h, and then stained with a 0.5% filtered Oil Red O (ORO) solution (Sigma-Aldrich, St. Louis, MI, USA) in 60% isopropanol and then washed with PBS. The lipid accumulation analysis was extracted by incorporating the ORO dye using isopropanol and then measuring at 490 nm using a PowerwaveTM XS microplate spectrophotometer (Agilent, Santa Clara, CA, USA). The cell images were detected with a microscope (CKX41, Olympus, Tokyo, Japan) with a digital camera (UCMOS05100KPA, ToupTek, Hangzhou, Zhejiang, China).

### 4.6. Immunofluorescence Staining

Mature adipocytes (day 12) were fixed with 4% paraformaldehyde and washed with PBS, and then permeabilization and blocking were performed with PBS containing 0.3% Triton X-100 and 3% BSA. These cells were then incubated with the primary antibody anti-UCP1 (A5857, diluted 1:500, ABclonal Inc., Woburn, MA, USA) at 4 °C overnight. After PBS washing, the cells were incubated with the secondary antibody Alexa Fluor 488 (AS053, diluted 1:1000, ABclonal Inc., Woburn, MA, USA) for 90 min at room temperature in the dark. The cell images were observed through a fluorescence microscope (IX71) with a digital camera (DP71) (both from Olympus, Tokyo, Japan) after 4′,6-diamidino-2-phenylindole (DAPI) (Vector Laboratories Inc., Burlingame, CA, USA) staining.

### 4.7. MitoTracker Staining

To stain the mitochondria, a pre-warmed staining solution containing 200 nM MitoTracker Red probe (Thermo Fisher Scientific, Waltham, MA, USA) was added to the mature adipocytes (day 12) followed by incubation at 37 °C for 40 min in a humidified atmosphere with 5% CO_2_, and the cell images were observed with a fluorescence microscope (IX71) with a digital camera (DP71).

### 4.8. Mitochondrial DNA Copy Number Determination

The total DNA of differentiated hADSCs, for 12 days, was isolated using a genomic DNA isolation kit (Bioneer, Daejeon, Korea) according to the manufacturer’s instructions, and the mitochondrial DNA copy number performed and analyzed the relative expression ratio of the mitochondrial-encoded gene (mt-RNR2) and nuclear-encoded gene (B2M) using a Step One Plus Real-Time PCR system (Thermo Fisher Scientific, Waltham, MA, USA).

### 4.9. Statistical Analysis

All data are presented as means ± standard error of the mean (SEM), and the unpaired two-tailed Student’s *t*-test was performed to compare the two groups. The differences between groups were analyzed through Tukey’s one-way analysis of variance (ANOVA) or a two-way ANOVA using GraphPad Prism 6 (San Diego, CA, USA), and a *p* < 0.05 was considered significant.

## Data Availability

All raw data used for the figure generations in this manuscript can be obtained by contacting the corresponding author.

## Supplementary Materials

The following supporting information can be downloaded at: https://www.mdpi.com/article/10.3390/ijms23126764/s1.

## Abbreviations

ADIPOQ: adiponectin, C1Q and collagen domain-containing; CAR4, carbonic anhydrase 4; CEBPA, CCAAT/enhancer-binding protein alpha; CIDEA, cell death-inducing DNA fragmentation factor, alpha subunit-like effector A; CIDEC, cell death-inducing DFFA-like effector C; CITED1, Cbp/p300-interacting transactivator with Glu/Asp-rich carboxy-terminal domain 1; COX7A1, cytochrome c oxidase subunit 7A1; CPT1B, carnitine palmitoyltransferase 1B; CPT2, carnitine palmitoyltransferase 2; CYCS, cytochrome c; DIO2, deiodinase, iodothyronine, type II; DNM2, dynamin 2; ELOVL3, elongation of very-long-chain fatty acid-like 3; FABP3, fatty acid-binding protein 3; FASN, fatty acid synthase; MFF, mitochondrial fission factor; MFN1, mitofusin 1; MFN2, mitofusin 2; PAT2, proton-coupled amino acid transporter 2; PPARG, peroxisome proliferator-activated receptor gamma; PPARGC1A, peroxisome proliferator-activated receptor alpha; SLC25A20, solute carrier family 25 member 20; TFAM, mitochondrial transcription factor A.

## References

[1] Rosen E.D., Spiegelman B.M. (2014). What we talk about when we talk about fat. Cell.

[2] Ikeda K., Maretich P., Kajimura S. (2018). The common and distinct features of brown and beige adipocytes. Trends Endocrinol. Metab..

[3] Chu D.-T., Gawronska-Kozak B. (2017). Brown and brite adipocytes: Same function, but different origin and response. Biochimie.

[4] Seale P., Bjork B., Yang W., Kajimura S., Chin S., Kuang S., Scime A., Devarakonda S., Conroe H.M., Erdjument-Bromage H. (2008). PRDM16 controls a brown fat/skeletal muscle switch. Nature.

[5] Sidossis L.S., Porter C., Saraf M.K., Børsheim E., Radhakrishnan R.S., Chao T., Ali A., Chondronikola M., Mlcak R., Finnerty C.C. (2015). Browning of subcutaneous white adipose tissue in humans after severe adrenergic stress. Cell Metab..

[6] Kajimura S., Spiegelman B.M., Seale P. (2015). Brown and beige fat: Physiological roles beyond heat generation. Cell Metab..

[7] Paulo E., Wang B. (2019). Towards a better understanding of beige adipocyte plasticity. Cells.

[8] Arner P., Bernard S., Salehpour M., Possnert G., Liebl J., Steier P., Buchholz B.A., Eriksson M., Arner E., Hauner H. (2011). Dynamics of human adipose lipid turnover in health and metabolic disease. Nature.

[9] Lee J.H., Park A., Oh K.-J., Lee S.C., Kim W.K., Bae K.-H. (2019). The role of adipose tissue mitochondria: Regulation of mitochondrial function for the treatment of metabolic diseases. Int. J. Mol. Sci..

[10] Sanchis-Gomar F., Derbré F. (2014). Mitochondrial fission and fusion in human diseases. N. Engl. J. Med..

[11] Boutant M., Kulkarni S.S., Joffraud M., Ratajczak J., Valera-Alberni M., Combe R., Zorzano A., Cantó C. (2017). Mfn2 is critical for brown adipose tissue thermogenic function. EMBO J..

[12] Mahdaviani K., Benador I.Y., Su S., Gharakhanian R.A., Stiles L., Trudeau K.M., Cardamone M., Enríquez-Zarralanga V., Ritou E., Aprahamian T. (2017). Mfn2 deletion in brown adipose tissue protects from insulin resistance and impairs thermogenesis. EMBO Rep..

[13] Bean C., Audano M., Varanita T., Favaretto F., Medaglia M., Gerdol M., Pernas L., Stasi F., Giacomello M., Herkenne S. (2021). The mitochondrial protein Opa1 promotes adipocyte browning that is dependent on urea cycle metabolites. Nat. Metab..

[14] de Mello A.H., Costa A.B., Engel J.D.G., Rezin G.T. (2018). Mitochondrial dysfunction in obesity. Life Sci..

[15] Cogliati S., Frezza C., Soriano M.E., Varanita T., Quintana-Cabrera R., Corrado M., Cipolat S., Costa V., Casarin A., Gomes L.C. (2013). Mitochondrial cristae shape determines respiratory chain supercomplexes assembly and respiratory efficiency. Cell.

[16] Klionsky D.J., Abdel-Aziz A.K., Abdelfatah S., Abdellatif M., Abdoli A., Abel S., Abeliovich H., Abildgaard M.H., Abudu Y.P., Acevedo-Arozena A. (2021). Guidelines for the use and interpretation of assays for monitoring autophagy. Autophagy.

[17] Jang J.Y., Blum A., Liu J., Finkel T. (2018). The role of mitochondria in aging. J. Clin. Investig..

[18] Lu X., Altshuler-Keylin S., Wang Q., Chen Y., Henrique Sponton C., Ikeda K., Maretich P., Yoneshiro T., Kajimura S. (2018). Mitophagy controls beige adipocyte maintenance through a Parkin-dependent and UCP1-independent mechanism. Sci. Signal..

[19] Vámos A., Shaw A., Varga K., Csomós I., Mocsár G., Balajthy Z., Lányi C., Bacso Z., Szatmári-Tóth M., Kristóf E. (2022). Mitophagy Mediates the Beige to White Transition of Human Primary Subcutaneous Adipocytes Ex Vivo. Pharmaceuticals.

[20] Ashrafi G., Schlehe J.S., LaVoie M.J., Schwarz T.L. (2014). Mitophagy of damaged mitochondria occurs locally in distal neuronal axons and requires PINK1 and Parkin. J. Cell Biol..

[21] Koyano F., Okatsu K., Kosako H., Tamura Y., Go E., Kimura M., Kimura Y., Tsuchiya H., Yoshihara H., Hirokawa T. (2014). Ubiquitin is phosphorylated by PINK1 to activate parkin. Nature.

[22] Pickles S., Vigié P., Youle R.J. (2018). Mitophagy and quality control mechanisms in mitochondrial maintenance. Curr. Biol..

[23] Wiedemann N., Kozjak V., Chacinska A., Schönfisch B., Rospert S., Ryan M.T., Pfanner N., Meisinger C. (2003). Machinery for protein sorting and assembly in the mitochondrial outer membrane. Nature.

[24] Kozjak V., Wiedemann N., Milenkovic D., Lohaus C., Meyer H.E., Guiard B., Meisinger C., Pfanner N. (2003). An essential role of Sam50 in the protein sorting and assembly machinery of the mitochondrial outer membrane. J. Biol. Chem..

[25] Ding C., Wu Z., Huang L., Wang Y., Xue J., Chen S., Deng Z., Wang L., Song Z., Chen S. (2015). Mitofilin and CHCHD6 physically interact with Sam50 to sustain cristae structure. Sci. Rep..

[26] Ott C., Ross K., Straub S., Thiede B., Götz M., Goosmann C., Krischke M., Mueller M.J., Krohne G., Rudel T. (2012). Sam50 functions in mitochondrial intermembrane space bridging and biogenesis of respiratory complexes. Mol. Cell. Biol..

[27] Xu R., Le Kang S.W., Yang C., Fu Y., Ding Z., Zou Y. (2021). Samm50 Promotes Hypertrophy by Regulating Pink1-Dependent Mitophagy Signaling in Neonatal Cardiomyocytes. Front. Cardiovasc. Med..

[28] Jian F., Chen D., Chen L., Yan C., Lu B., Zhu Y., Chen S., Shi A., Chan D.C., Song Z. (2018). Sam50 regulates PINK1-Parkin-mediated mitophagy by controlling PINK1 stability and mitochondrial morphology. Cell Rep..

[29] Moffat J., Grueneberg D.A., Yang X., Kim S.Y., Kloepfer A.M., Hinkle G., Piqani B., Eisenhaure T.M., Luo B., Grenier J.K. (2006). A lentiviral RNAi library for human and mouse genes applied to an arrayed viral high-content screen. Cell.

[30] Sarbassov D.D., Guertin D.A., Ali S.M., Sabatini D.M. (2005). Phosphorylation and regulation of Akt/PKB by the rictor-mTOR complex. Science.

[31] Jeong J.-Y., Yim H.-S., Ryu J.-Y., Lee H.S., Lee J.-H., Seen D.-S., Kang S.G. (2012). One-step sequence-and ligation-independent cloning as a rapid and versatile cloning method for functional genomics studies. Appl. Environ. Microbiol..

[32] Hill J.O., Wyatt H.R., Peters J.C. (2012). Energy balance and obesity. Circulation.

[33] Yu R., Lendahl U., Nistér M., Zhao J. (2020). Regulation of mammalian mitochondrial dynamics: Opportunities and challenges. Front. Endocrinol..

[34] Michurina S., Stafeev I., Menshikov M., Parfyonova Y.V. (2021). Mitochondrial dynamics keep balance of nutrient combustion in thermogenic adipocytes. Mitochondrion.

[35] Hung C.H.-L., Cheng S.S.-Y., Cheung Y.-T., Wuwongse S., Zhang N.Q., Ho Y.-S., Lee S.M.-Y., Chang R.C.-C. (2018). A reciprocal relationship between reactive oxygen species and mitochondrial dynamics in neurodegeneration. Redox Biol..

[36] Harms M.J., Li Q., Lee S., Zhang C., Kull B., Hallen S., Thorell A., Alexandersson I., Hagberg C.E., Peng X.-R. (2019). Mature human white adipocytes cultured under membranes maintain identity, function, and can transdifferentiate into brown-like adipocytes. Cell Rep..

[37] Xue R., Lynes M.D., Dreyfuss J.M., Shamsi F., Schulz T.J., Zhang H., Huang T.L., Townsend K.L., Li Y., Takahashi H. (2015). Clonal analyses and gene profiling identify genetic biomarkers of the thermogenic potential of human brown and white preadipocytes. Nat. Med..

[38] Chen Y., Dorn G.W. (2013). PINK1-phosphorylated mitofusin 2 is a Parkin receptor for culling damaged mitochondria. Science.

[39] Pryde K.R., Smith H.L., Chau K.-Y., Schapira A.H. (2016). PINK1 disables the anti-fission machinery to segregate damaged mitochondria for mitophagy. J. Cell Biol..

[40] Yu J., Zhang S., Cui L., Wang W., Na H., Zhu X., Li L., Xu G., Yang F., Christian M. (2015). Lipid droplet remodeling and interaction with mitochondria in mouse brown adipose tissue during cold treatment. Biochim. Biophys. Acta (BBA)-Mol. Cell Res..

