# Supplementary material for: SAMM50 Regulates Thermogenesis of Beige Adipocytes Differentiated from Human Adipose-Derived Stem Cells by Balancing Mitochondrial Dynamics
Source: Int J Mol Sci. 2022 Jun 17;23(12):6764. doi: 10.3390/ijms23126764 (PMC9224253; doi:10.3390/ijms23126764)
Supplement: Supplementary file 1 [file ijms-23-06764-s001.zip › ijms-1727102-supplementary.pdf]

Supplementary Materials

Supplemental Table S1

Table S1. The used primer sequences for quantitative PCR (5'-3').

Gene	Forward	Reverse	Accession no.
GAPDH	GGAAGGTGAAGGTCGGAGTC	GAAGGGGTCATTGATGGCAAC	NM_001256799
CEBPA	GTCGGTGGACAAGAACAGCAA	CATTGTCACTGGTCAGCTCCA	NM_001279
PPARG	GACCCAGAAAGCGATTCCTTC	TCCATTACGGAGAGATCCACG	NM_001330615
ADIPOQ	TTGCCTACCACATCACAGTCT	TTACGCTCTCCTTCCCCATAC	NM_001177800
FASN	TCAGCCGCCATCTACAACATC	CAGCACCACATCCTCAAACAC	NM_004104
UPC1	ACTTGGTGTCGGCTCTTATCG	CCGTTGGTCCTTCGTTAGTGA	NM_021833
SAMM50	CTGTCAAGTGGGAAGGCGTAT	ATGGCGTGCGAAAGAGATGAT	NM_015380
PPARGC1A	ACCCACCACTCCTCCTCATAA	GTCTTCCTTTCCTCGTGTCCA	NM_001330751
DIO2	GTCCTCCATCAGGTTTTAGCAA	CTCACCCAATTTCACCATCCA	NM_000793
ELOVL3	CAGTGAGGATGTGGGGCATTA	ATGATGAAGGCTGTGTCTCCG	NM_152310
CIDEA	TGGGAGACAACACGCATTTCA	TCATACATGGTGGCCTTCACG	NM_001279
CIDEC	AAGTCCCTTAGCCTTCTCTACC	CCTTCCTCACGCTTCGATCC	NM_001199551
PAT2	GACATCAAGGCCAGCATAAGC	GACGAGGCGAATGGACAGAT	NM_181776
SLC25A20	AGACACAGCCACCGAGTTTG	TCCCCAAACCAAACCCAAAGA	NM_000387
FABP3	ACCAAGCCTACCACAATCATCG	CAAGTTTCCCTCCATCCAGTGT	NM_001320996
PDK4	TCAGCCTTCCCTTACACCAAT	AAACCAGCCAAAGGAGCATTC	NM_002612
CITED1	CCTCACCTGCGAAGGAGGA	GGAGAGCCTATTGGAGATCCC	NM_001144885
CYCS	TGGGTGATGTTGAGAAAGGCA	TGGCGGCTGTGTAAGAGTATC	NM_018947
COX7A1	GAGTGCGCGAGAAACAGAAG	CCCAGCCAAGGGAGTACAAG	NM_001864
TFAM	ATGGCGTTTCTCCGAAGCAT	TCCGCCCTATAAGCATCTTGA	NM_001270782
CPT1B	ACCAGGATCTGGGCTATGTGT	TACCGCTGAATTGTGGCTGAC	NM_001145134
CPT2	CAGCAGCCAAAGGGATCATCT	TACCCAACACCAAAGCCATCA	NM_000098
MFN1	AAATGCTCAAAGGGTGCTCCT	AGATGTAACGGACGCCAATCC	NM_033540
MFN2	CTGTCTGGGACCTTTGCTCAT	TGCAGGTACTGGTGTGTGAAC	NM_001127660
OPA1	AAGGGTCTGCTTGGTGAGCT	CTTGCGCTTCTGTTGGGCATA	NM_001354663
DNM1L	TGCCGTGAACCTGCTAGATG	GCCTTTGGCACACTGTCTTG	NM_001278463
DNM2	CCCCGACATGGCCTTTGAAG	GAGCTTCTCGGCACACTTTT	NM_001005360
MFF	GCCTCATCAGTCCACCCAAC	CTGGCACCAGACCATGACTT	NM_001277061

Supplemental Table S2

Table S2. The used shRNA sequences for knock down of SAMM50.

Clone ID	Target sequences	Oligonucleotide sequence
TRCN000 0229422	5′-CATCGCTCG GTTGGAACTTAA-3′	F: 5′-CCGGCATCGCTCGGTTGGAACTTAACTCGAGTTAAGTTCCAACC GAGCGATGTTTTTG-3′<br>R: 5′-AATTCAAAAACATCGCTCGGTTGGAACTTAACTCGAGTTAAGTT CCAACCGAGCGATG-3′
TRCN000 0229420	5′-GAGATTAAC GGGCAGTTATAA-3′	F: 5′-CCGGGAGATTAACGGGCAGTTATAACTCGAGTTATAACTGCCC GTTAATCTCTTTTTG-3′<br>R: 5′-AATTCAAAAAGAGATTAACGGGCAGTTATAACTCGAGTTATAA CTGCCCGTTAATCTC-3′

Supplemental Figure S1

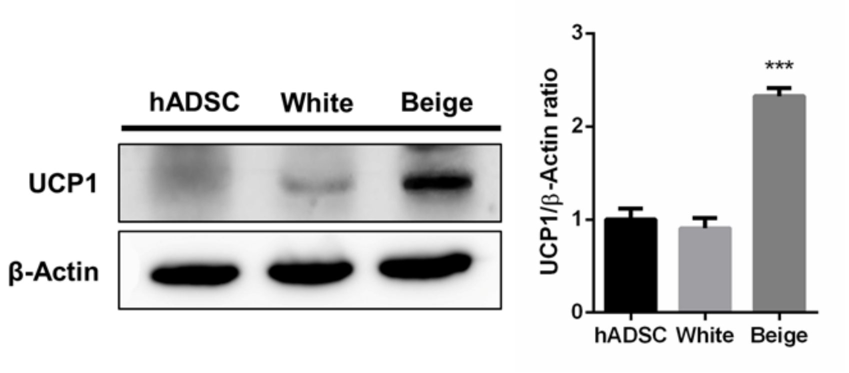

Figure S1. Western blot analysis was performed to examine the protein levels of UCP1 in the human adipose-derived stem cells, white adipocytes, and beige adipocytes. The protein levels of UCP1 was quantified using ImageJ software. Results are shown as mean ± SEM (n = 3). *, $p < 0.05$; **, $p < 0.01$; ***, $p < 0.005$ compared to the hADSC and White groups.

Supplemental Figure S2

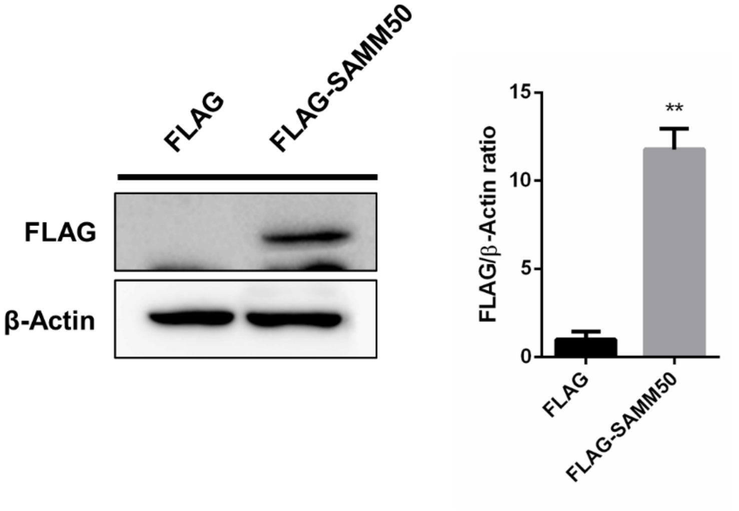

Figure S2. Western blot analysis was performed to examine the protein levels of FLAG in the FLAG and the human adipose-derived stem cells infected FLAG-tagged SAMM50 using lentivirus. The protein levels of FLAG was quantified using ImageJ software. Results are shown as mean ± SEM (n = 3). *, $p < 0.05$; **, $p < 0.01$; ***, $p < 0.005$ compared to the FLAG group.
